# The role of maize sap beetles (Coleoptera: Nitidulidae) and maize weevils (Coleoptera: Curculionidae) in the spread of *Aspergillus flavus* in pre-harvest maize in Kenya

**DOI:** 10.1093/jee/toae217

**Published:** 2024-10-09

**Authors:** Ginson Riungu, James W Muthomi, Wolfgang Buechs, John M Wagacha, Esther Sheila Philip, Torsten Meiners

**Affiliations:** Sugar Research Institute, Kenya Agricultural and Livestock Research Organization, Kisumu, Kenya; Department of Plant Science and Crop Protection, Faculty of Agriculture, University of Nairobi, Nairobi, Kenya; Department of Plant Science and Crop Protection, Faculty of Agriculture, University of Nairobi, Nairobi, Kenya; Institute for Biology and Chemistry, University of Hildesheim, Hildesheim, Germany; Department of Biology, Faculty of Science and Technology, University of Nairobi, Nairobi, Kenya; Kenya Plant Health Inspectorate Service, Phytosanitary Section, Nairobi, Kenya; Julius Kuehn Institute, Institute for Ecological Chemistry, Plant Analysis and Stored Product Protection, Berlin, Germany

**Keywords:** *Aspergillus*, *Carpophilus*, maize weevil, aflatoxin, pre-harvest maize

## Abstract

The spread of toxigenic *Aspergillus* into maize by insects and the subsequent aflatoxin contamination poses a risk to humans and animals and has been investigated in North and South America. To evaluate this effect in an African context, Greenhouse studies were conducted in 2022 to determine the role of sap beetles, *Carpophilu*s *dimidiatus* Fabricius, 1792 (Coleoptera: Nitidulidae) and maize weevils, *Sitophilus zeamais* Motschulsky, 1855 (Coleoptera: Curculionidae) on infection of maize kernels by *Aspergillus flavus* Link and the resultant aflatoxin accumulation. To test the beetles’ efficacy, treatments were applied on partially opened primary ears at 3 different stages of kernel development (BBCH 75, 83, and 87). The treatments were: (i) distilled water, (ii) water with *A. flavus* spores, (iii) maize grits, (iv) maize grits with *A. flavus* spores, (v) *C. dimidiatus*, (vi) *C. dimidiatus* with *A. flavus* spores, (vii) *S. zeamais*, and (viii) *S. zeamais* with *A. flavus* spores. Data on kernel infection, maize rotting, yield, and aflatoxin content in kernels were collected. The highest kernel spoilage and yield loss were recorded for the co-inoculation of *S. zeamais* and *A. flavus* spore*s*, followed by *S. zeamais* without *A. flavus* spores, and then *C. dimidiatus* with the fungal spores. Inoculation of maize at the BBCH 83 growth stage resulted in the highest kernel damage and aflatoxin contamination. *S. zeamais* and, to a lesser extent, *C. dimidiatus* effectively spread the *A. flavus* inoculum into non-wounded ears, resulting in fungal and aflatoxin contamination. The yield loss from *S. zeamais*-*Aspergillus* co-inoculation occurred due to the grain rotting and actual feeding of the maize weevils. Thus, insect management is important in reducing pre-harvest contamination of maize with mycotoxigenic fungi and their resultant toxins.

## Graphical Abstract

**Graphical Abstract:**
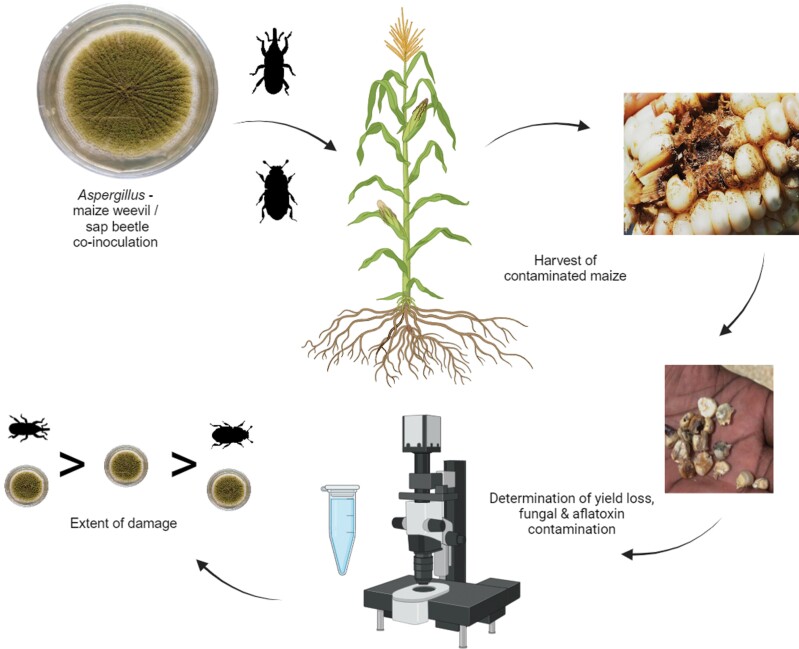

## Introduction

Maize (*Zea mays* L.) is a staple food for many households in East Africa, a key ingredient in animal feed, and is used to manufacture various industrial products. However, the mycotoxin contamination of maize used for food and feed in Kenya and most sub-Saharan African countries remains a challenge (Probst et al. 2014, Mwihia et al. 2020). The contamination is mainly attributed to infection by *Aspergillus flavus*, which produces aflatoxins (Udoh et al. 2000, Muthomi et al. 2012), or *Fusarium verticillioides* (Sacc.) Nirenberg, which produces fumonisins (Giorni et al. 2019). High levels of aflatoxin in maize have been recorded in the Eastern region of Kenya, an occurrence that is attributed to the prevailing climatic conditions (Probst et al. 2014, Mutunga et al. 2017) and the presence of a highly virulent and toxigenic strain of *A. flavus*, the S-morphotype (Probst et al. 2014).

Ingestion of high levels of aflatoxins may lead to acute aflatoxicosis and death (Lewis et al. 2005), whereas chronic exposure to low doses predisposes humans to liver cancer and may affect protein metabolism and immunity, and thus may worsen disease infections and malnutrition (Williams et al. 2004). Chronic exposure to aflatoxins is associated with impaired growth in children (Kang’ethe et al. 2017, Mahfuz et al. 2018) and with hepatocellular carcinoma (HCC), particularly in individuals chronically infected with the hepatitis B virus (Mitchell et al. 2017).

In addition to the health concerns for consumers, aflatoxin contamination lowers the market value and uses for the contaminated crop. Animals fed with contaminated maize or its byproducts may exhibit stunted growth and reduced yields of milk and eggs (Schmale and Munkvold 2009, Suleiman et al. 2015).

Pre-harvest entry of fungal pathogens into maize occurs through the silks and later infection into glumes, kernels, ear tips, and down to the ear base (Koehler 1942, Marsh and Payne 1984, Thompson and Raizada 2018). The fungal spores are carried into the entry points mainly by lepidopterans and coleopterans (Drepper and Renfro 1990, Nesci and Etcheverry 2002, Chulze 2010). Among the caterpillars associated with the enhancement of mycotoxins in maize are the corn earworm (*Helicoverpa zea* Boddie), European corn borer (*Ostrinia nubilalis* Hübner), and the fall armyworm (*Spodoptera frugiperda*, J. E. Smith), whereas among the beetles, maize weevils (*Sitophilus zeamais* Motschulsky) (Coleoptera: Curculionidae), and sap beetles (*Carpophilus* spp. Stephens) (Coleoptera: Nitidulidae), are important (Dowd, 1998). Other insects, including moths, worms, bees, grasshoppers, and flies, are often colonized by *Aspergillu*s and other mycotoxigenic fungi and can, therefore, vector them to maize and other crops (Guthrie et al. 1982, Bosque-Pérez and Mareck 1991, St Leger et al. 2000, Gupta and Gopal 2002).

In previous studies, wounding and non-wounding techniques were applied when inoculating maize with *Aspergillus*. Commonly used non-wounding techniques include spraying and atomization of spores onto maize silks (Payne et al. 1988) and the use of granular material infected with *A. flavus* to inoculate ears (Olanya et al. 1997, Bock and Cotty 1999). Spray inoculation has been used to study *A. flavus* interaction with southwestern corn borer (*Diatraea grandiosella*, Dyar), and the interaction significantly increased aflatoxin contamination (Windham et al. 1999). Wounding inoculation techniques used in maize trials include the pinbar, knife, pinboard, side needle, toothpick, and punch drill (Windham et al. 2003).

In a recent study, *C. dimidiatus* and *S. zeamais* collected from maize in 3 regions in Kenya were colonized with aflatoxigenic strains of *A. flavus* and *A. minisclerotigenes* Vaamonde (Riungu et al., personal observation). Sap beetles, including *Carpophilus lugubris* Murray and *C. freemani* Dobson, have been identified as vectors of *Aspergillus flavus* to maize and can contaminate maize with aflatoxins (Lussenhop and Wicklow 1990). Though listed as secondary pests, sap beetles have, in some instances, caused substantial damage to maize by feeding on damaged or exposed kernels where they feed on fungi growing on the wounds or directly on the kernels, thus enhancing mycotoxins contamination (Bruck et al. 2006). In contrast, *S. zeamais* is a major pest of maize, particularly in storage, and damages maize, leading to quality and quantity losses (Khakata et al. 2018), including mycotoxin contamination (Beti et al. 1995).

However, little is known about the effect of insect infestation on the spread of the fungal inoculum in maize before harvest, particularly in Kenya and Africa, where maize is a staple food. Most studies have been conducted on stored maize, and when done on maize ears, the wounding method has been used (Windham et al. 2003, 2018). Trials mimicking the vectoring aspect without wounding the ears and the stage of kernel development when the co-occurrence would be most detrimental in maize are scarce. Therefore, the present study investigated (1) the impact of *S. zeamais* and *C. dimidiatus* in the spread of *Aspergillus* spores in maize, (2) the resulting fungal infection and aflatoxin contamination, and (3) the stage of kernel development that is the most suitable for fungal inoculation.

## Materials and Methods

### Experimental Site and Crop Establishment

Experiments were conducted in 2 greenhouse cycles set up in April and October 2022 to correspond with Kenya’s long and short rain cropping seasons, respectively. Maize was sown in an insect-proof greenhouse at the Kibos Sugar Research Institute in Kisumu County, Kenya (GPS coordinates: N 00° 02ʹ 11″, E 34° 49ʹ 17″, 1,250 m above sea level). It has an annual average temperature of 19.7 °C, an average relative humidity of 66%, and an average annual precipitation of 1,362 mm (Nyberg et al. 2020). The area experiences a bimodal rainfall pattern from March to July and September to January for long and short rains, respectively. The maize hybrid WH 505 (FAO 500, Western Seed Company, Kenya), which matures in 120–130 days, has a level 3 husk coverage on a 1 to 5 scale, where 1 = very loose, 2 = loose, 3 = moderate tightness, 4 = tight, and 5 = very tight, and is commonly grown in Western Kenya was used. It was grown in 15 cm diameter plastic buckets filled with forest soil obtained from the Kakamega forest and sterilized by autoclaving at 121 °C to ensure no *Aspergillus* spores were present. To each pot, 5 g of di-ammonium phosphate fertilizer (18:46:0) was added.

### Insect Rearing, Inoculum Preparation, and Inoculation

Sap beetle (*C. dimidiatus*) and maize weevils (*S. zeamais*) were collected from maize fields in Makueni County, Kenya, and identified using morphological characteristics (Tumuramye 2010, Reales et al. 2018) with the help of Mr. Morris Mutua, an entomology expert at the National Museum of Kenya. Voucher specimens were deposited at the Kenya Agricultural and Livestock Research Organization (KALRO). Cultures of *C. dimidiatus* were maintained in sterile fresh maize ears placed in 3-L glass jars with the bottom layered with 2 cm moist sterile soil. The ears were obtained from the KALRO biotechnology laboratory and were replaced weekly to ensure constant nutrition. The jars were incubated at 30 ± 2°C, 72% ± 10% RH, and a photoperiod of 12:12 h (L:D). Pre-adult stages and young adult *C. dimidiatus* were fed on a sterilized maize meal prepared using a coffee grinder (AR1100, Moulinex, UK) and placed on 9 cm diameter Petri dishes (Odeyemi et al. 2004). The Petri dishes were kept in conditions similar to the jars for maintaining the adults above. The maize for preparing the meal had been grown in a greenhouse at the research center. The maize meal had been autoclaved and samples were plated on agar plates to confirm it had no *Aspergillus* contamination.

Maize weevils (*S. zeamais)* were reared by placing 20 pairs of 1-week-old beetles in 2 kg of sterile maize placed in 5 kg capacity Kilner jars covered with mesh lids (Ojo and Amoloye 2016). The jars were then incubated for 7 days at 26 ± 0.5 °C, 55% ± 5% RH, and 12:12 h (L:D) photoperiod (Power and Pritam 1974) until inoculation. An aflatoxigenic strain of *A. flavus* (MKU-6) isolated from *C. dimidiatus* obtained from naturally infected maize ears from Makueni County, Kenya, was used in the trials. The identity of the fungus was confirmed based on cultural and morphological features (Klich 2002), spore characteristics observed under a Motic BA410 microscope (Meyer Instruments, Inc, Houston, USA), and by earlier DNA sequencing (Riungu et al., not published). Spores were multiplied on sterile maize grits (40 mesh, Ramtons Grinder RM541, autoclaved at 121 °C for 15 min). Two cork borer discs of a 5-day *A. flavus* colony were placed into the maize grits (100 mL distilled water, 50 g of maize grits into a 500-mL flask) and incubated at 28 ± 2 °C (Windham et al. 1999). After 7 days, a gram of the maize grits was placed in 25-mL plastic sterile centrifuge tubes, and in each tube, 5 adults of either the *C. dimidiatus* or the *S. zeamais* were introduced. Beetles were placed in centrifuge tubes containing sterile maize grits for the uninoculated control. The tubes were plugged using a sterile cotton plug, shaken, and left for 1 h for the spores to come into contact with the beetles’ cuticles (Bhusal and Khanal 2019). The inoculated vs. uninoculated beetles were placed on partially opened primary ears to expose the silks. The ears were then covered with muslin cloths to contain the insects, and the whole crops in different batches were randomly placed in mesh fabric cages (BugDorm 6M630, MegaView Science Co., Ltd., Taiwan) in the greenhouse to reduce the risk of cross-contamination.

The treatments entailed either spraying maize ears with (i) distilled water, (ii) distilled water with *A. flavus spores*, or placing (iii) maize grits, (iv) maize grits with *A. flavus* spores, (v) *C. dimidiatus* without *A. flavus* spores, (vi) *C. dimidiatus* with *A. flavus* spores, (vii) *S. zeamais* without *A. flavus* spores, and (viii) *S. zeamais* with *A. flavus* spores.

The treatments were applied on partially opened primary ears to expose the silks at 3 different times of kernel development: the milky stage, BBCH 75; early dough stage, BBCH 83; and at physiological maturity, BBCH 87. A 1-L garden flower sprayer was used to spray the distilled water treatments.

### Harvesting, Sample Handling, and Grain Data Collection

Harvesting was done at BBCH 97, and from each batch, 5 ears were harvested and de-husked for ear rot rating assessment on a scale of 1 to 5, based on a visual appraisal of grain color and development, where 1 = sound and 5 = kernels damaged and covered with the fungus or discolored (Cardwell et al. 2000). The other batch was shelled by hand, and kernels sundried to a moisture content of below 13.5% (Twist Grain Pro, Draminski, Poland) and finely milled using a coffee and spice grinder (AR1100, Moulinex, UK) for aflatoxin analysis and fungal enumeration. The blender was disassembled, brushed, and rinsed between samples using 70% ethanol.

The 400 seeds’ weight was determined using a weighing balance (Nimbus 1602E, Adam Equipment, UK), and the grains were sorted into clean vs unusable/spoilt grains. A kernel was described as spoilt if rated at 4 or 5 on the rotting scale described above. The damaged and undamaged grains were counted and weighed separately in 4 runs of 100 seeds, and the yield loss was determined using the function:


$$\begin{array}{l} \%\;yield\;loss=\;\frac{weight\;of\;spoilt\;grain}{weight\;of\;400\;seeds}\times 100 \end{array}$$

Fungal contamination was determined by diluting 1 g of flour into 9 mL of distilled water successively and plating the10^-−3^ dilutions in 3 replicates on Potato Dextrose Agar modified with an antibiotic (39 g PDA, oxoid, 25 mg chloramphenicol, in a liter of water. The plates were incubated for 7 days at 28 °C, and yellow–green-colored colonies characteristic of *A. flavus* were enumerated, and the mean from the 3 plates was expressed as colony-forming units per gram (CFU/g) of flour. The identity of the *Aspergillus* was confirmed using the morphological features described by Klich (2002).

Total aflatoxin was analyzed on 10 g of flour in duplicates using the manufacturer’s instructions (Helica, Biosystems Inc.). The assay is based on a solid-phase competitive inhibition enzyme immunoassay with an aflatoxin-specific antibody optimized to cross-react with all 4 subtypes of aflatoxin (B1, B2, G1, and G2) in grain with a detection limit of 20 ppb (Hygiena 2023).

### Data Analysis

Data were subjected to SAS version 9.4 (SAS Institute, Cary, NC 2001) for analysis of variance (ANOVA) at *P* ≤ 0.05. A combined analysis of variance was carried out on all the collected data to determine the effects of the cycles, treatment, development stages, and their interactions. Data for the weight of rotten grains, percent infection rate, yield loss, CFU/g, and aflatoxins (ppb) were not normally distributed, and therefore they were log-transformed (log_10_). Since the factor cycle did not influence the data in the ANOVA, we performed a combined analysis.

The mean separation test was performed using Tukey’s honestly significant difference (Tukey HSD) procedure at *P* ≤ 0.05 level of significance for each trait determined whenever the main effects were significant.

## Results

### Effect of *Aspergillus* and Insect Treatments on Cob and Kernel Damage and Yield Parameters

**Fig. 1.**
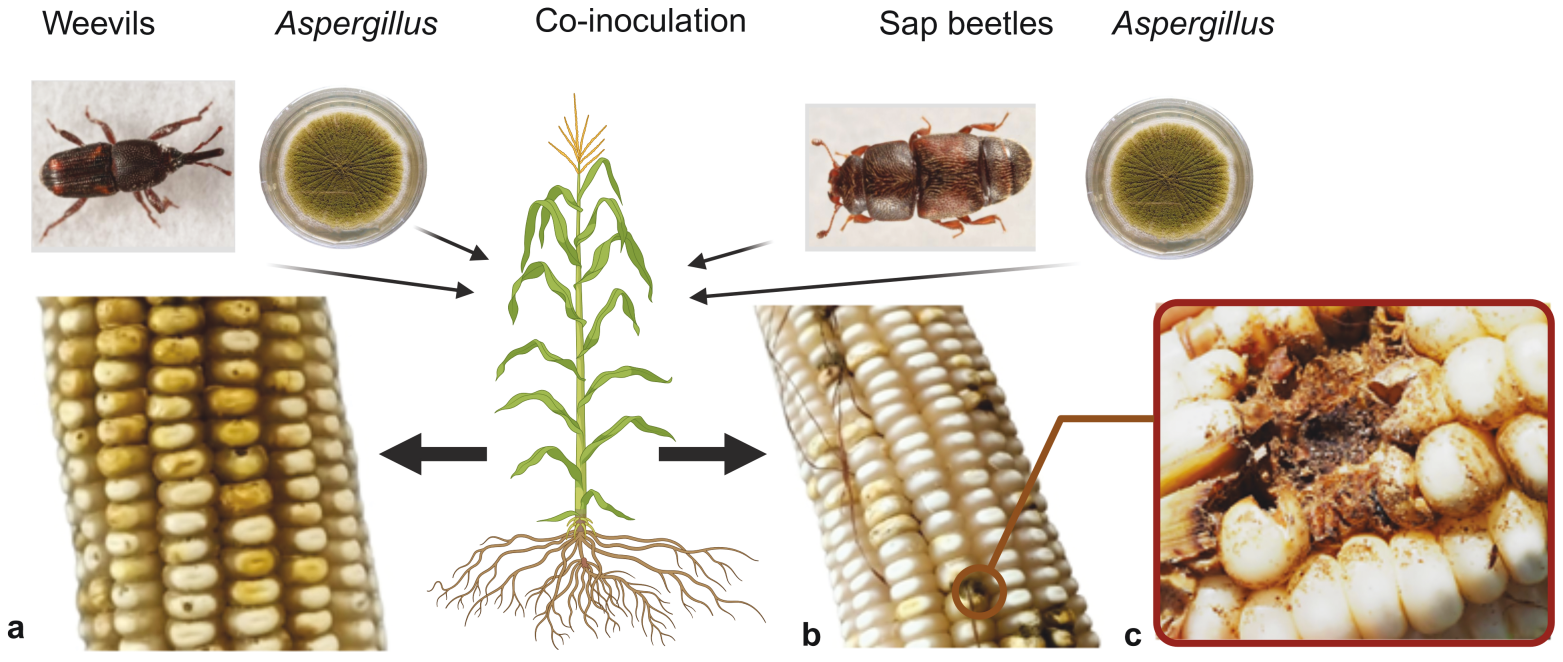
Damage after co-inoculation of *Aspergillus* and insects. A) Damage after co-inoculation with *S. zeamais*; B) and C) damage after co-inoculation with *C. dimidiatus*.The figure was created with BioRender.com.

**Fig. 2.**
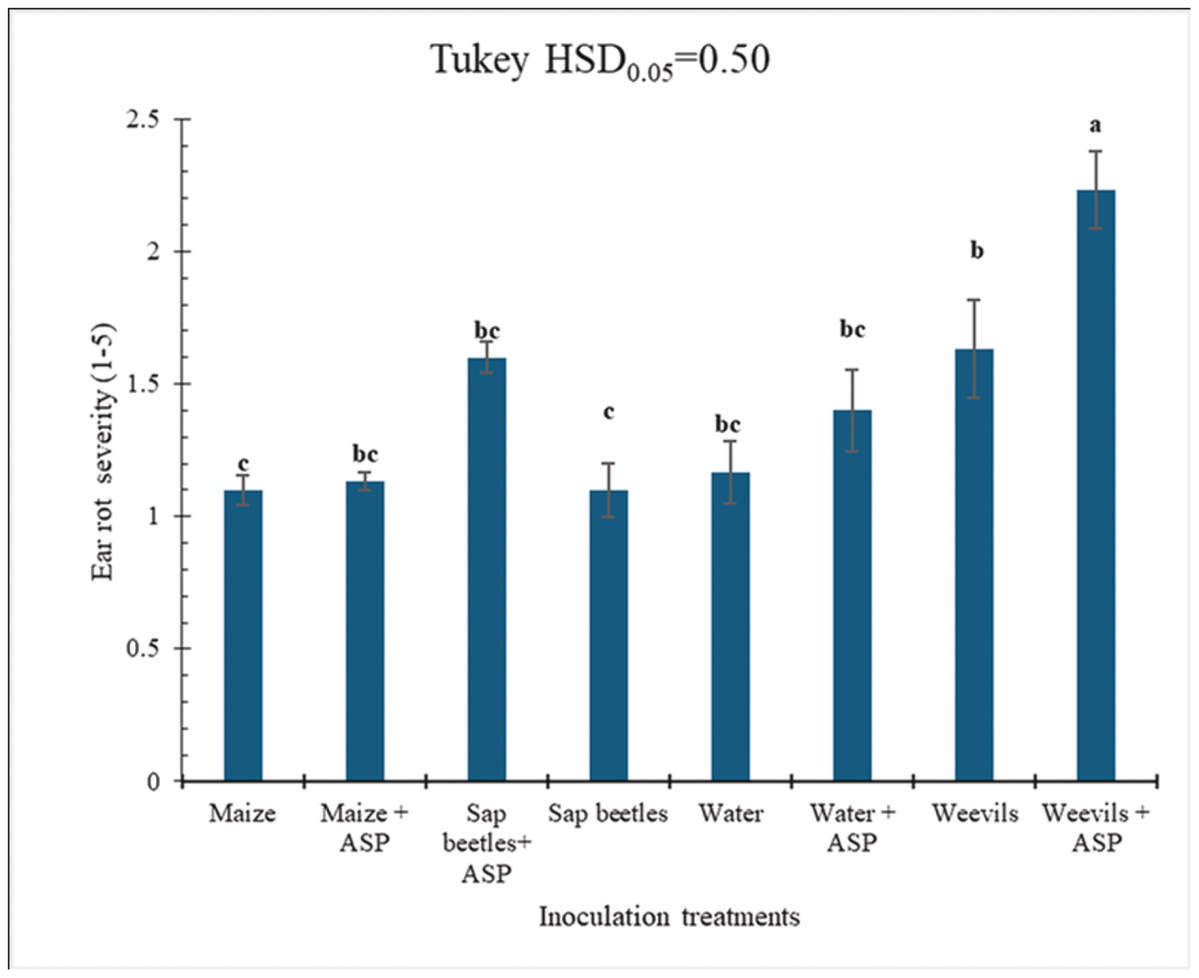
Mean ear rot severity (scales 1–5) after inoculating maize ears with water, maize grits, *S. zeamais*, and *C. dimidiatus* with or without *A. flavus* (ASP). Values on the *y*-axis are means, and error bars represent the standard error of the mean; the *x*-axis is inoculation treatments; bar graphs accompanied by the same letters are not significantly different (Tukey’s honestly significant difference tests, *P* ≤ 0.05).

The co-inoculation of maize with *Aspergillus-S. zeamais* or *Aspergillus*-*C. dimidiatus* resulted in visible rotting and seed damage. The rots and damage were more pronounced in ears co-inoculated with *Aspergillus* and *S. zeamais*, which showed the weevil entry and exit holes on the seeds (Fig. 1A). In contrast, the *C. dimidiatus*-*Aspergillus* treated ears had some rotting but no exit holes (Fig. 1B and C). Within a range from 1 to 5, the combined mean of ear rot severity from the 2 experimental cycles ranged from 1.1 in the control to 2.2 in the weevil-*Aspergillus* co-inoculation treatments (Fig. 2). Analysis of variance showed that treatment, time, and their interactions significantly (*P* ≤ 0.05) affected many of the parameters (Table 1).

**Table 1.** ANOVA results on the influence of cycle, treatment (water, maize grits, *S. zeamais*, and *C. dimidiatus* with or without *A. flavus* [ASP]), and plant development stage/time (*T*) on the number and weight of clean seeds, kernel infection rate, weight of rotten grain, weight of 400 kernels, yield loss, fungal contamination, and aflatoxin contamination

Effects	df	No. clean seeds/400		Weight clean seed (g)		Infection rate %		Weight rotten seed (g)	
		F-value	*P*-value	F-value	*P*-value	F-value	*P*-value	F-value	*P*-value
Cycle (S)	1	2.95	0.0878	0.37	0.5465	4.33	0.0392	1.5	0.2228
Treatment (TRT)	7	26.21	<0.0001	5.23	<0.0001	26.41	<0.0001	19.9	<0.00001
Time (*T*)	2	13.56	<0.0001	0.17	0.8397	9.44	0.0001	14.42	<0.0001
Replicates	3	0.38	0.7655	1.01	0.3892	0.7	0.5507	0.66	0.581
S × TRT	7	0.65	0.7121	0.23	0.977	0.62	0.7405	0.82	0.5723
S × T	2	0.37	0.6886	1.82	0.166	0.02	0.9819	0.26	0.7696
TRT × T	14	7.09	<0.0001	2.91	0.0007	2.78	0.0011	5.58	<0.0001
S × TRT × T	14	0.63	0.8344	0.34	0.9866	0.65	0.8214	0.68	0.7905
Residual Mean square	141		219.68		439.88		0.15		0.03
*R* ^*2*^			0.7		0.39		0.65		0.65
C.V (%)			3.84		17.28		13.13		7.1

Effects	df	Weight 400 seeds (g)		Yield loss %		CFU/g		Aflatoxin level (ppb)	
		*F*-value	*P*-value	*F*-value	*P*-value	*F*-value	*P*-value	*F*-value	*P*-value
Cycle (S)	1	0.14	0.704	1.76	0.1863	4.95	0.0277	0.61	0.4344
Treatment (TRT)	7	3.71	0.001	22.82	<0.0001	19.19	<0.0001	467.5	<0.0001
Time (T)	2	0.87	0.4223	12.25	<0.0001	7.28	0.001	1.68	0.1892
Replicates	3	0.85	0.4673	0.87	0.4575	0.85	0.4696	1.3	0.2763
S × TRT	7	0.19	0.9881	0.8	0.5884	0.51	0.8275	0.69	0.6845
S × T	2	2.02	0.1361	0.16	0.8541	0.21	0.8147	0.31	0.7364
TRT × T	14	3.1	0.0003	4.87	<0.0001	1.54	0.1053	1.81	0.0418
S × TRT × T	14	0.35	0.9848	0.66	0.8123	0.63	0.8324	0.81	0.6532
Residual Mean square	141		458.75		0.02		0.71		0
*R* ^*2*^			0.37		0.66		0.58		0.96
C.V (%)			17.32		6.17		19.81		2.51

df, degrees of freedom; R2, coefficient of determination; CV (%), coefficient of variation. The weight of rotten seed, infection rate %, yield loss %, colony-forming units/g (CFU/g), and aflatoxin level (ppb) were log-transformed.

The treatment significantly affected the number of clean seeds (*F* = 26.21; df = 7, 14; *P* < 0.001). The highest number of clean seeds was recorded in ears treated with distilled water (397.4 ± 0.1), and the lowest (349.6 ± 4.6) was in seeds with interaction between *S. zeamais* and *Aspergillus* (Table 2). Similarly, the weight of the clean seed was significantly influenced by the treatments (*F* = 5.23; df = 7, 14; *P* < 0.0001), with the heaviest clean seed (137.0 ± 1.8 g) reported from maize grit treated seeds while the lowest weight (110.0 ± 2.5 g) was reported from the *S. zeamais-Aspergillus* interaction. The weight of rotten seed was significantly influenced only by the interaction of *S. zeamais* and the pathogen (*F* = 19.9; df = 7, 14; *P* < 0.001) to record the highest weight of (9.2 ± 1.1 g). The infection rate varied across the treatments (F = 26.41; df = 7, 14; *P* < 0.001), with the *S. zeamais-Aspergillus* treated seeds recording the highest infection rate of (45.6% ± 3.8 %). The *S. zeamais-Aspergillus* interaction was the only treatment that resulted in significant yield loss (*F* = 22.82; df = 7, 14; *P* < 0.001), of (7.5% ± 0.8%) and aflatoxin levels (*F* = 467.5; df = 7, 14; *P* < 0.001) of 12.1 ppb total aflatoxin content.

**Table 2.** Effect of treatment (water, maize grits, *S. zeamais*, and *C. dimidiatus* with or without *A. flavus* [ASP]) on the number and weight of clean seeds, kernel infection rate, weight of rotten grain, weight of 400 kernels, yield loss, fungal, and aflatoxin contamination

Treatments	No. clean seeds/400	Weight clean seed (g)	Infection rate %	Weight rotten seed (g)	Weight.400 seeds (g)	Yield loss %	CFU/g	Aflatoxin level (ppb)
Maize grits	394.6 ± 0.5a	137.0 ± 1.8a	5.4 ± 0.5c	0.7 ± 0.1b	137.7 ± 1.9a	0.5 ± 0.1b	44.6 ± 4.7c	<LOD
Maize grits + ASP	389.5 ± 1.8ab	121.8 ± 2.5abc	10.5 ± 1.9bc	1.8 ± 0.4b	123.6 ± 2.5ab	1.4 ± 0.3b	96.2 ± 16.6bc	<LOD
*C. dimidiatus*	393.6 ± 0.4ab	117.3 ± 1.6bc	6.4 ± 0.4c	1.0 ± 0.1b	118.3 ± 1.7bc	0.8 ± 0.1b	57.4 ± 3.8c	<LOD
*C. dimidiatus *+ ASP	388.8 ± 1.5ab	118.5 ± 2.7abc	11.2 ± 1.5bc	2.0 ± 0.4b	120.5 ± 2.9abc	1.4 ± 0.2b	100.5 ± 13.3bc	<LOD
*S. zeamais*	381.3 ± 0.9b	113.1 ± 1.7c	18.7 ± 0.9b	2.3 ± 0.2b	115.4 ± 1.7c	2.1 ± 0.2b	168.0 ± 8.0b	<LOD
*S. zeamais* + ASP	349.6 ± 4.6c	110.0 ± 2.5c	45.6 ± 3.8a	9.2 ± 1.1a	119.2 ± 2.8abc	7.5 ± 0.8a	414.2 ± 34.8a	12.1
Water	397.4 ± 0.1a	135.3 ± 1.7ab	2.6 ± 0.1c	0.6 ± 0.1b	135.9 ± 1.7ab	0.4 ± 0.0b	23.6 ± 1.3c	<LOD
Water + ASP	393.2 ± 0.4ab	118.0 ± 2.7bc	6.8 ± 0.4c	0.8 ± 0.1b	118.8 ± 2.7abc	0.7 ± 0.1b	61.1 ± 3.5bc	<LOD
Tukey HSD _0.05_	13.2	18.6	11.8	3.0	19.0	2.2	107.1	

This means that a column followed by the same letter(s) is not significantly different (Tukey’s honestly significant difference test, *P* ≤ 0.05).

LOD, limit of detection, CFU/g, colony-forming units per gram.

### Effect of Time of Inoculation on Kernel Damage, Grain Yield, Fungal, and Aflatoxin Contamination

The time of inoculation significantly affected the number of clean seeds (*F* = 13.56; df = 2, 14; *P* < 0.0001), the infection rate (*F* = 26.41; df = 2, 14; *P* < 0.0001), the weight of rotten seeds (*F* = 19.9; df = 2, 14; *P* < 0.0001), % yield loss (*F* = 12.25; df = 2, 14; *P* < 0.0001), and fungal contamination of seeds measured as colony-forming units per gram of seeds (CFU/g; *F* = 7.28; df = 2, 14; *P* < 0.001; Table 1). Inoculation at BBCH 83 (early dough stage) resulted in a higher seed infection rate, yield loss, and colony-forming units compared to inoculations at BBCH 75 (milky stage) and BBCH 87 (physiological maturity). The number of clean seeds ranged from 378.4 ± 3.5 (BBCH83) to 391.5 ± 1.0 (BBCH87), while the infection rate ranged from 8.5% ± 1.0% (BBCH87) to 19.8% ± 3.0 % (BBCH83). The weight of rotten seeds ranged from 1.1 ± 0.2 g (BBCH87) to 4.1 ± 0.8 g (BBCH83; Table 3). The aflatoxin levels were not affected by inoculation time.

**Table 3.** Effect of inoculation time (treatment with water, maize grits, *S. zeamais*, and *C. dimidiatus* with or without *A. flavus* [ASP] at 3 kernel development stages [BBCH 75, 83, and 87]) on the number and weight of clean seeds, kernel infection rate, weight of rotten grain, weight of 400 kernels, yield loss, fungal, and aflatoxin contamination

Kernel development stages	No. clean seeds/400	Weight clean seed (g)	Infection rate %	Weight rotten seed (g)	Weight 400 seeds (g)	Yield loss %	CFU/g	Aflatoxin level (ppb)
BBCH75	388.2 ± 1.7a	120.2 ± 2.6a	11.8 ± 1.7b	1.7 ± 0.2b	121.9 ± 2.6a	1.6 ± 1.7b	105.7 ± 14.9b	1.7 ± 0.2a
BBCH83	378.4 ± 3.5b	122.4 ± 2.2a	19.8 ± 3.0a	4.1 ± 0.8a	126.5 ± 2.3a	3.0 ± 3.0a	179.9 ± 27.0a	1.9 ± 0.5a
BBCH87	391.5 ± 1.0a	121.6 ± 2.2a	8.5 ± 1.0b	1.1 ± 0.2b	122.6 ± 2.2a	0.9 ± 1.0b	76.5 ± 9.0b	1.5 ± 0.4a
Tukey HSD _0.05_	6.2	8.8	5.6	1.4	9.0	1.1	50.5	0.4

Means followed by the same letter within columns are not significantly different (Tukey’s honestly significant difference test, *P* ≤ 0.05). CFU/g, colony-forming units per gram.

## Discussion

This is the first study in Kenya and Africa to document the significant role of *S. zeamais* and *C. dimidiatus* in spreading *Aspergillus* and the resultant aflatoxin contamination in pre-harvest maize. The interaction of these beetles with *Aspergillus* on maize kernels increased the *Aspergillus* infection rate, kernel rotting, grain yield loss, and total aflatoxin contamination in maize. The method of inoculation or infestation and the timing of inoculation or infestation are critical for these effects. A synergistic relationship was found between *A. flavus* treatment and the presence of *S. zeamais*, resulting in a tenfold increase in infection rate and aflatoxin contamination in maize. These findings are congruent with earlier studies showing positive correlations between insect damage and aflatoxin content in maize kernels (Rodriguez-del-Bosque 1996, Ni et al. 2011). In addition, our study demonstrates for the first time that the beetle interaction with *A. flavus* on previously non-wounded kernels leads to considerable infection and aflatoxin contamination, indicating that mechanical wounding by beetle feeding activity is the entry point for the fungus.

The higher *Aspergillus* and aflatoxin contamination in maize co-inoculated with *S. zeamais* and *Aspergillus* could be because weevils are primary pests in maize and are able to cause entry points on the maize reducing quantity and quality of maize while enhancing aflatoxin and other mycotoxins contamination (Khakata et al. 2018). Insect damage can enhance aflatoxin levels in pre-harvest maize either by acting as vectors, by facilitating fungal spore entry, or by damaging the kernel pericarp, thus increasing infection (Widstrom 1979, Xu et al. 2022) or from the metabolic activity of the insects that increase the moisture content or temperatures of the grains (Bhusal and Khanal 2019). In this study, the untreated weevils significantly raised the ear rotting, suggesting that the metabolic activity of the weevils coupled with the damage to kernels, even in the absence of *Aspergillus*, can lead to rotting and quality deterioration of kernels.

In studies on stored grains, Lemic et al. (2021) reported higher mechanical damage and aflatoxin levels when *Aspergillus* was co-inoculated with either the granary weevil (*S. granarius*) or the maize weevil (*S. zeamais*). The authors confirmed earlier reports of *S. zeamais* enhancing aflatoxins in stored maize by over 10% (Bhusal and Khanal 2019).

Co-inoculation of *A. flavus* with insects (*C. dimidiatus* or *S. zeamais*) was most effective in enhancing *Aspergillus* infection while using dry maize grits was the least effective method of inoculating the pathogen. The method of inoculation is known to affect the level of infection and the resultant toxin accumulation (Widstrom et al. 1981). Ni et al. (2011) studied the spatial pattern of natural infestation of corn earworms (*H. zea*), fall armyworms (*S. frugiperda*), maize weevils (*S. zeamais*), and brown stinkbugs (*Euschistus servus* Say) and the corresponding aflatoxin contamination under field conditions in the United States. They reported that the toxin levels correlated better with kernel-feeding pre-harvest by *S. zeamais* and kernel damage by stinkbugs than with silk- and cob-feeding damage by earworms and the fall armyworm. The authors suggested that kernel feeding at pre-harvest was more important than silk- and cob-feeding damage by lepidopteran pests post-flowering. We hypothesize from our study that weevil damage created entry points for the *A. flavus* (Ni et al. 2011, Louis et al. 2015, García-Díaz et al. 2020) and, coupled with the vectoring of spores to the kernels, increased fungal colonization and aflatoxin content at harvest.

The *S. zeamais* enhanced aflatoxin contamination to 12 ppb, a level above the 10 ppb acceptable limit for total aflatoxins in maize for human consumption. The ingestion of high levels of aflatoxins can result in aflatoxicosis and fatalities, as has been reported in Kenya before (Lewis et al. 2005). Chronic exposure to aflatoxins, on the other hand, is linked to impaired growth in children and ailments, including cancer (Kang’ethe et al. 2017).

McMillan et al. (1987) studied the impact of husk cover and species of infesting insects, and similar to this study, *S. zeamais* had the highest enhancement of aflatoxin contamination and yield loss. Demissie et al. (2008) reported that the husk cover influenced field infestation of maize by *S. zeamais* in Bako, Western Ethiopia. Although the *C. dimidiatus* infestation slightly increased yield loss and aflatoxin contamination in the kernels, it was not as high as in weevil-infested ears. This highlights the importance of *C. dimidiatus* as a pest in maize production. Although they are labeled secondary pests that mainly colonize damaged ears, sap beetles are important vectors of mycotoxigenic fungi, particularly *Aspergillus* and *Fusarium* (Dowd 1995). Moreover, the nitidulids have been implicated as spore vectors for *Aspergillus* (Lussenhop and Wicklow 1990), *F. moniliforme* Sheldon (Chalivendra et al. 2020), and *F. graminearum* Schwabe (Attwater and Busch 1983) to pre-harvest maize ears. Considering that the infection and aflatoxin contamination rates were lower than that of the *S. zeamais* in the current study, we hypothesize that their role is mainly vectoring with minor kernel damage.

The low contamination with *Aspergillus* and aflatoxins from the interaction between *Aspergillus* and *C. dimidiatus* as compared to that between the fungus and *S. zeamais* could be attributed to the fact that maize is most appealing to sap beetles just after silking and again at post-physiological maturity of maize (Fleischer et al. 2021), which was outside the 3 development stages tested. In addition, sap beetles are primarily secondary pests attracted to insect and plant volatiles emitted from damage by primary pests, and the damaged ears act as entry points for the beetles (Fleischer et al. 2021). In our case, we used a non-wounding method, which might have made the ears less attractive to the beetles and denied the *C. dimidiatus* entry points. Currently, the fall armyworms (*S. frugiperda*) and other caterpillars heavily damage maize in farmer fields; and could be the reason the *C. dimidiatus* has become an important pest and fungal vector in maize. *C. dimidiatus* was used in our study due to its abundance on Kenyan maize farms. Our results indicate that it is not very destructive on its own. The sap beetle used in the study is very small compared to those that have been previously reported as fungal vectors to maize in the US. Larger sap beetles do a lot more damage to the kernels themselves, as do the larvae resulting from eggs laid in ears by the adults.

Barry et al. (1985) tested the efficacy of the wheat curl mite *Eriophyes tulipae* (Kifer) and the maize weevil *Sitophilus zeamais* as vectors of *A. flavus* in maize kernels under field conditions in Missouri, USA. The mite was not an effective vector of *A. flavus* spores, whereas *S. zeamais* dusted with the spores effectively vectored the spores in areas with irregular natural fungi levels.

Aflatoxin contamination was highest under the *Aspergillus-S. zeamais* interaction. Aflatoxin is produced when the fungus invades the maize embryo or endosperm, which explains the random pattern of the toxin’s occurrence even among kernels from the same ear (Smart et al. 1990). In our study, the non-wounding of kernels could have hindered the entry of the fungal hyphae into the embryo and endosperm, resulting in low aflatoxin contamination.

Introducing *Aspergillus* at the early dough stage (BBCH 83) was most effective for disease development. Widstrom et al. (1981), who noted that the highest infection and aflatoxin content in kernels was when treatments were done 20 days after silking, reported similar results. Marsh and Payne (1984) used the silk colors (green–yellow, yellow–brown, and brown) as the silk stages. They indicated that inoculations at the yellow–brown silks resulted in the highest fungal and aflatoxin contamination. They attributed it to the diminished defense at senescence and the availability of high levels of nutrients for fungal growth.

This study demonstrates the importance of timing inoculation in trials with *A. flavus* with or without insect infestation. When the fungus was applied at BBCH 83, kernel infection, rotting, yield loss, and aflatoxin contamination were generally higher than when applied at BBCH 73 and BBCH 87. Penetration of fungal hyphae into seeds is hindered by the testa, which thickens as the grains mature, except over the embryo. This could explain the comparatively low contamination of maize with *A. flavus* at physiological maturity (BBCH 87).

The damaged ears and kernels act as fungal entry points (Widstrom 1979, Hutchison et al. 2010). Widstrom (1979) indicated that the principal role of insects in enhancing mycotoxin contamination in kernels is by predisposing plant tissues to the fungus. The corn borer, earworms, and fall armyworms are the main insects that enhance *Aspergillus* infection and the resultant toxins’ contamination in pre-harvest maize. In the current study, the damage of *S. zeamais* to the kernels seems to have created entry points more effectively than *C. dimidiatus*, hence the higher contamination with *A. flavus* and the aflatoxins. Zuber (1977) showed that *Aspergillus* was a weak pathogen of maize, and a successful grain infection required a break of the pericarp (Guo et al. 1993).

The current study showed that *S. zeamais* and *C. dimidiatus* can effectively vector *A. flavus* into pre-harvest maize, with *S. zeamais* being more effective than *C. dimidiatus*. It also revealed that the timing and method of application of *A. flavus* greatly influence disease development and the resultant aflatoxin contamination in maize kernels. Considering integrated pest management our study suggests that greater efforts in insect pest management can play a role in reducing aflatoxin contamination in maize. Strategies such as intercropping and push-pull, combined with a careful use of insecticides that minimize insect infestation would significantly reduce *Aspergillus* infection and aflatoxin contamination. Similar tests should be undertaken to determine the efficacy of other arthropod taxa in vectoring *Aspergillus* spores. Tests on the effect of wounding maize ears before co-inoculating the fungus and the arthropods are also recommended.
